# Supplementary material for: The Relationship between Character Traits and In Vivo Cerebral Serotonin Transporter Availability in Healthy Subjects: A High-Resolution PET Study with C-11 DASB
Source: Pharmaceuticals (Basel). 2023 May 18;16(5):759. doi: 10.3390/ph16050759 (PMC10224397; doi:10.3390/ph16050759)
Supplement: Supplementary file 1 [file pharmaceuticals-16-00759-s001.zip › pharmaceuticals-2387410-supplementary.pdf]

Supplementary Materials

Supplementary Table S1. Comparison between males and females for raw scores of three character traits

Three character scales of TCI	Male (n = 12)	Female (n = 12)	p value
Self-directedness	58.2 ± 6.9	51.3 ± 7.1	0.024*
Cooperativeness	59.1 ± 8.4	59.9 ± 7.7	0.803
Self-transcendence	20.5 ± 13.7	24.9 ± 13.2	0.430

The raw scores of three character traits were presented as mean ± standard deviation, and p values were obtained using independent samples t-tests. In this table, an asterisk indicates statistical significance at $p < 0.05$. TCI, Temperament and Character Inventory.

Supplementary Table S2. Comparison between males and females for regional [11C]DASB BP_{ND} values

Brain region		Male (n = 12)	Female (n = 12)	*p* value
Frontal lobe	Rt. superior frontal gyrus	0.28 ± 0.05	0.26 ± 0.04	0.218
	Lt. superior frontal gyrus	0.26 ± 0.05	0.24 ± 0.04	0.136
	Rt. middle frontal gyrus	0.28 ± 0.06	0.26 ± 0.05	0.498
	Lt. middle frontal gyrus	0.28 ± 0.05	0.27 ± 0.05	0.601
	Rt. superior frontal gyrus (medial)	0.26 ± 0.04	0.26 ± 0.04	0.870
	Lt. superior frontal gyrus (medial)	0.25 ± 0.05	0.23 ± 0.03	0.411
	Rt. superior frontal gyrus (orbital part)	0.32 ± 0.05	0.31 ± 0.06	0.530
	Lt. superior frontal gyrus (orbital part)	0.30 ± 0.06	0.25 ± 0.05	0.049*
	Rt. superior frontal gyrus (medial orbital)	0.38 ± 0.06	0.38 ± 0.06	0.921
	Lt. superior frontal gyrus (medial orbital)	0.34 ± 0.09	0.34 ± 0.04	0.909
	Rt. middle frontal gyrus (orbital part)	0.29 ± 0.07	0.26 ± 0.05	0.163
	Lt. middle frontal gyrus (orbital part)	0.25 ± 0.04	0.23 ± 0.05	0.284
	Rt. inferior frontal gyrus (orbital part)	0.30 ± 0.07	0.30 ± 0.06	0.813
	Lt. inferior frontal gyrus (orbital part)	0.29 ± 0.04	0.30 ± 0.06	0.540
	Rt. olfactory cortex	1.08 ± 0.22	0.96 ± 0.15	0.153
	Lt. olfactory cortex	1.02 ± 0.20	0.94 ± 0.14	0.281
Temporal lobe	Rt. superior temporal gyrus	0.35 ± 0.03	0.33 ± 0.04	0.082
	Lt. superior temporal gyrus	0.47 ± 0.05	0.44 ± 0.07	0.294
	Rt. middle temporal gyrus	0.31 ± 0.04	0.26 ± 0.03	0.001**
	Lt. middle temporal gyrus	0.41 ± 0.06	0.39 ± 0.05	0.396

	Rt. inferior temporal gyrus	0.36 ± 0.05	0.29 ± 0.04	<0.001†
	Lt. inferior temporal gyrus	0.43 ± 0.04	0.39 ± 0.06	0.062
Parietal lobe	Rt. superior parietal gyrus	0.24 ± 0.03	0.22 ± 0.05	0.210
	Lt. superior parietal gyrus	0.23 ± 0.04	0.23 ± 0.05	0.832
	Rt. inferior parietal gyrus	0.22 ± 0.04	0.22 ± 0.04	0.700
	Lt. inferior parietal gyrus	0.28 ± 0.05	0.31 ± 0.06	0.141
Occipital lobe	Rt. middle occipital gyrus	0.28 ± 0.05	0.24 ± 0.05	0.108
	Lt. middle occipital gyrus	0.32 ± 0.05	0.31 ± 0.06	0.754
Limbic lobe	Rt. anterior cingulate & paracingulate gyri	0.52 ± 0.06	0.48 ± 0.07	0.139
	Lt. anterior cingulate & paracingulate gyri	0.52 ± 0.09	0.50 ± 0.05	0.472
	Rt. median cingulate & paracingulate gyri	0.55 ± 0.05	0.51 ± 0.04	0.089
	Lt. median cingulate & paracingulate gyri	0.55 ± 0.06	0.51 ± 0.04	0.065
	Rt. posterior cingulate gyrus	0.37 ± 0.08	0.33 ± 0.06	0.180
	Lt. posterior cingulate gyrus	0.42 ± 0.06	0.41 ± 0.07	0.835
	Rt. hippocampus	0.89 ± 0.13	0.74 ± 0.13	0.014*
	Lt. hippocampus	1.02 ± 0.09	0.89 ± 0.16	0.027*
Rt. Insula		0.63 ± 0.08	0.59 ± 0.09	0.374
Lt. Insula		0.71 ± 0.08	0.63 ± 0.11	0.059
Subcortical gray nuclei	Rt. amygdala	1.57 ± 0.27	1.38 ± 0.23	0.074
	Lt. amygdala	1.92 ± 0.35	1.84 ± 0.29	0.560
	Rt. caudate nucleus	1.17 ± 0.36	1.11 ± 0.26	0.606
	Lt. caudate nucleus	1.25 ± 0.39	1.10 ± 0.25	0.278

	Rt. putamen	1.40 ± 0.27	1.22 ± 0.20	0.079
	Lt. putamen	1.51 ± 0.38	1.40 ± 0.13	0.343
	Rt. nucleus accumbens	1.75 ± 0.31	1.59 ± 0.28	0.217
	Lt. nucleus accumbens	1.64 ± 0.35	1.44 ± 0.24	0.125
	Rt. globus pallidus	0.82 ± 0.28	0.82 ± 0.23	0.982
	Lt. globus pallidus	0.57 ± 0.22	0.62 ± 0.15	0.557
	Rt. thalamus	1.62 ± 0.29	1.72 ± 0.30	0.426
	Lt. thalamus	1.57 ± 0.23	1.62 ± 0.28	0.625
Brainstem	Dorsal raphe nucleus	1.70 ± 1.08	1.35 ± 0.40	0.312
	Median raphe nucleus	1.95 ± 1.20	2.16 ± 1.52	0.716

[^{11}C]DASB BP_{ND} values were indicated as mean ± standard deviation, and p values were calculated using independent samples t-tests. In these analysis, the statistical significance is indicated as $p < 0.05$*, $p < 0.01$**, and $p < 0.001^{\dagger}$. BP_{ND}, binding potential; Rt, right; Lt, left.

Supplementary Table S3. Significant correlations between the character traits and [^{11}C]DASB BP_{ND} in the brain regions excluded from the ROIs

Brain region		[^{11}C]DASB BP_{ND} value (Mean ± SD)	Correlation coefficient (p value)		
			Self-directedness	Cooperativeness	Self-transcendence
Central region	Rt. postcentral gyrus	0.28 ± 0.04	0.154 (0.494)	-0.095 (0.674)	-0.488 (0.021)*
Parietal lobe	Lt. angular gyrus	0.30 ± 0.05	0.429 (0.046)*	0.066 (0.771)	-0.014 (0.950)
	Rt. supramarginal gyrus	0.25 ± 0.05	-0.056 (0.805)	0.146 (0.516)	-0.441 (0.040)*
	Lt. supramarginal gyrus	0.37 ± 0.06	0.447 (0.037)*	0.200 (0.373)	0.208 (0.352)
Occipital lobe	Lt. inferior occipital gyrus	0.31 ± 0.06	0.498 (0.018)*	-0.128 (0.571)	0.044 (0.845)
	Rt. calcarine fissure and surrounding cortex	0.51 ± 0.09	0.591 (0.004)**	0.154 (0.495)	0.261 (0.242)
	Lt. calcarine fissure and surrounding cortex	0.47 ± 0.08	0.460 (0.031)*	0.020 (0.931)	0.013 (0.956)
	Rt. lingual gyrus	0.39 ± 0.08	0.451 (0.035)*	0.002 (0.995)	0.189 (0.400)
Thalamic nuclei	Rt. anteroventral nucleus	0.50 ± 0.18	-0.165 (0.462)	0.448 (0.037)*	0.213 (0.340)
	Lt. anteroventral nucleus	0.52 ± 0.20	-0.548 (0.008)**	0.211 (0.347)	-0.011 (0.961)
	Lt. ventral anterior	0.12 ± 0.07	-0.443 (0.039)*	0.342 (0.119)	-0.167 (0.457)
	Lt. intralaminar	0.29 ± 0.13	-0.243 (0.275)	-0.469 (0.028)*	-0.502 (0.017)*
Subcortical gray nuclei	Rt. red nucleus†	0.16 ± 0.13	0.557 (0.009)**	-0.215 (0.349)	-0.056 (0.809)

Correlation coefficients and p values were computed using partial correlation analysis with age and sex as covariates, and the statistical significance is presented as $p < 0.05$* and $p < 0.01$**. †[^{11}C]DASB BP_{ND} values in the right red nucleus were obtained from 23 healthy controls in the process of calculating these values using the SRTM2. BP_{ND}, binding potential; ROI, region of interest; SD, standard deviation; Rt, right; Lt, left; SRTM2, simplified reference tissue model 2.

Supplementary Table S4. Significant correlations between the character traits and [^{11}C]DASB BP_{ND} in ROIs for males and females separately

Sex	Brain region	[^{11}C]DASB BP_{ND} value (Mean ± SD)	Correlation coefficient (*p* value)		
			Self-directedness	Cooperativeness	Self-transcendence
Male (n = 12)	Rt. superior temporal gyrus	0.35 ± 0.03	-0.296 (0.377)	0.407 (0.215)	-0.707 (0.015)*
	Rt. middle temporal gyrus	0.31 ± 0.04	-0.224 (0.508)	0.381 (0.247)	-0.619 (0.042)*
	Rt. inferior temporal gyrus	0.36 ± 0.05	-0.130 (0.704)	-0.330 (0.322)	-0.611 (0.046)*
Female (n = 12)	Lt. superior frontal gyrus (medial orbital)	0.23 ± 0.03	0.609 (0.047)*	-0.026 (0.939)	0.114 (0.738)
	Lt. olfactory cortex	0.94 ± 0.14	0.615 (0.044)*	0.033 (0.923)	-0.110 (0.747)
	Lt. superior parietal gyrus	0.23 ± 0.05	0.663 (0.026)*	0.362 (0.274)	0.289 (0.389)
	Lt. inferior parietal gyrus	0.31 ± 0.06	0.681 (0.021)*	0.295 (0.379)	0.193 (0.570)
	Lt. hippocampus	0.89 ± 0.16	0.626 (0.039)*	0.251 (0.456)	-0.197 (0.561)
	Rt. Amygdala	1.38 ± 0.23	0.626 (0.039)*	-0.032 (0.926)	-0.047 (0.891)

Correlation coefficients and *p* values were obtained using partial correlation analysis with age as a covariate. Asterisks indicate statistical significance at $p < 0.05$. BP_{ND}, binding potential; ROI, region of interest; SD, standard deviation; Rt, right; Lt, left.

The relationship between the harm avoidance temperament and the 5-HTT availability in 52 a priori ROIs

The Temperament and Character Inventory was used to assess the harm avoidance temperament in 24 healthy subjects (12 males and 12 females). Based on a previous PET study [1], [^{11}C]DASB BP_{ND} values were obtained in 52 *a priori* ROIs involved in the serotonergic system. Based on several studies demonstrating age and sex effects on 5-HTT binding [2,3], a supplementary ROI-based partial correlation analysis with age and sex as covariates was performed using the Statistical Package for the Social Sciences (SPSS) v28.0 (IBM Corp., Armonk, NY, USA) to examine the relationship between the harm avoidance temperament and [^{11}C]DASB BP_{ND} in ROIs. Significant results were identified at a threshold of two-tailed $p < 0.05$.

The mean raw score for the harm avoidance temperament was 28.1 ± 7.1. The raw score of the harm avoidance temperament was not significantly correlated with age ($r = 0.25$, $p > 0.05$), and there was no significant difference in the harm avoidance temperament between males and females ($t = -1.63$, $p > 0.05$). The supplementary ROI-based analysis revealed that the harm avoidance temperament was significantly negatively correlated with [^{11}C]DASB BP_{ND} in the left posterior cingulate gyrus ($r = -0.468$, $p = 0.028$) (Supplementary Figure S1).

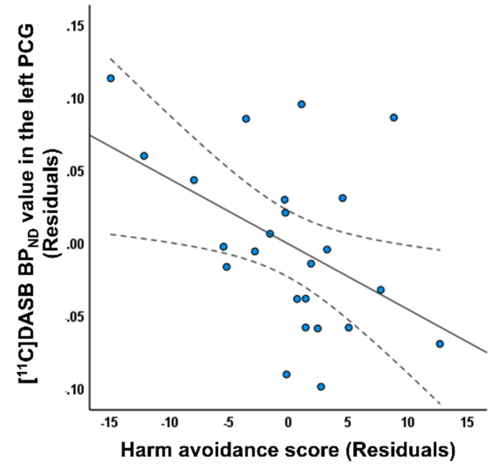

Supplementary Figure S1. Scatter plot showing a negative correlation between the raw score of the harm avoidance temperament and [^{11}C]DASB BP_{ND} in the left posterior cingulate gyrus controlling for age and sex ($r = -0.468$, $p = 0.028$). The solid and dotted lines represent the regression lines and 95% confidence intervals, respectively. BP_{ND}, binding potential; PCG, posterior cingulate gyrus.

[References]

1. Kim, J. H.; Kim, J. H.; Son, Y. D.; Joo, Y. H.; Lee, S. Y.; Kim, H. K.; Woo, M. K. Altered interregional correlations between serotonin transporter availability and cerebral glucose metabolism in schizophrenia: A high-resolution PET study using [C-11]DASB and [F-18]FDG. *Schizophr Res* **2017**, *182*, 55-65. DOI: 10.1016/j.schres.2016.10.020.

2. van Dyck, C. H.; Malison, R. T.; Seibyl, J. P.; Laruelle, M.; Klumpp, H.; Zoghbi, S. S.; Baldwin, R. M.; Innis, R. B. Age-related decline in central serotonin transporter availability with [I-123]beta-CIT SPECT. *Neurobiol Aging* **2000**, *21* (4), 497-501. DOI: 10.1016/S0197-4580(00)00152-4.
3. Jovanovic, H.; Lundberg, J.; Karlsson, P.; Cerin, A.; Saijo, T.; Varrone, A.; Halldin, C.; Nordstrom, A. L. Sex differences in the serotonin 1A receptor and serotonin transporter binding in the human brain measured by PET. *Neuroimage* **2008**, *39* (3), 1408-1419. DOI: 10.1016/j.neuroimage.2007.10.016.
